# KRAS-mutant colon cancer cells respond to combined treatment of ABT263 and axitinib

**DOI:** 10.1042/BSR20181786

**Published:** 2019-03-06

**Authors:** Guihua Wang, Ying Huang, Zhipeng Wu, Chunmei Zhao, Hui Cong, Shaoqing Ju, Xudong Wang

**Affiliations:** 1 Department of Laboratory Medicine Affiliated Hospital of Nantong University, 20 Xisi Road, Nantong 226001, JS, People’s Republic of China; 2 Department of Orthopedic Oncology, Changzheng Hospital, The Second Military Medical University, 415 Fengyang Road, Shanghai 200003, People’s Republic of China; 3 Clinical Tissue Bank, Department of Pathology, Affiliated Hospital of Nantong University, 20 Xisi Road, Nantong 226001, JS, People’s Republic of China

**Keywords:** ABT263, axitinib, combination treatment, combination index, KRAS-mutant colon cancer, synergy

## Abstract

Significant challenges to develop selective and effective pharmacological inhibitors for important oncoproteins like RAS continue impeding the success to treat cancers driven by such mutations. In the present study, the ABT263 and axitinib combination imposed synergistic effects on *RAS*-mutant colon cancer cells. The combination inhibited *in vitro* and *in vivo* growth of the cancer cells by enhancing apoptosis. Furthermore, AKT and Wnt/β-catenin signaling pathways were slightly down-regulated by the combination in *KRAS*-mutant colon cancer cells. The current results indicate that oncogene addiction can be targeted for therapy in colon cancer cells harboring the *RAS*-mutant. Therefore, targeting oncogene addiction can be a viable strategy for treating refractory cancers driven by important oncogenes, such as *KRAS*, which are otherwise difficult to be targeted by small molecules.

## Introduction

The pathogenesis of cancer involves a complicated multistep process where various mutagenic events endow the cancer cells with unique oncogenic features, including uncontrolled proliferation potential, self-sufficient growth, and resistance to apoptotic signals [1]. The complexity of highly varied genetic perturbations in cancer makes its treatment an intimidating task. One strategy to attack cancer is to target the critical hubs in the oncogenic signaling network to induce a systematic failure in the cancer cells [2]. However, to date, despite the discovery of multiple oncogenes and tumor suppressors such as p53, Ras, and p16^INK4a^, all the pharmacological efforts to target these critical proteins only meet limited success. The predicament may be ascribed to the significant challenges to develop selective and effective pharmacological inhibitors for proteins like p53 and RAS [3]. Overcoming these challenges, recent studies have explored new strategies by targeting oncogene/non-oncogene addiction for cancer therapy [4–11]. The underlying idea is that, a wide variety of genes and pathways can be targeted to induce synthetic lethality in a given tumor, because pairing the loss of function of one gene with that of a second gene may result in a severe and possibly lethal growth and survival phenotype, provided that proper target genes or pathways are identified [12].

We set out to pursue the strategy of oncogene/non-oncogene addiction to treat colon cancer cells in the current study. Colon cancer ranks the third most common cancer among both men and women in the United States and globally [13,14]. The mechanisms of the carcinogenesis of colon cancer are multifaceted and complicated. Genetic alterations in *APC, KRAS, TP53, SMAD4*, and *PIK3CA* comprise the majority of the genetic events found in colon cancer [14,15]. As a major isoform of RAS, KRAS mutations are frequently found in many cancers, including pancreatic, colon, and lung cancers. Despite decades of endeavors to target KRAS for cancer therapy, the efforts have not been very successful, partly due to the challenges to locate pharmacologically suitable docking site for small molecular drugs [2]. However, a recent synthetic lethal drug screening showed a proof-of-concept where combinatorial drug treatment can be an effective strategy to treat *RAS*-mutant cancer cells [3]. Instead of developing *RAS*-specific inhibitors, the components in the pathways that are upstream/downstream of RAS signaling, or in cross-talk with RAS signaling, can be targeted and coordinated to produce a profound pharmacological action on killing the *KRAS*-mutant tumors. Given the possible effects of synthetic lethality induced by oncogene addiction targeting [5–8,10,11], we reasoned that there exist synthetic lethal genes that can be targeted for the treatment of certain types of *RAS*-mutant colon cancer.

In the present study, we employ the combinatorial drugs targeting KRAS synthetic lethal genes in colon cancer cells; we found that ABT263 (a Bcl-xL inhibitor) and axitinib (AXIT), a tyrosine kinase inhibitor selective for vascular endothelial growth factor receptors [(VEGFR)-1, -2, and -3], showed cytotoxic effects. Following the discovery, to test the hypothesis that oncogene addiction can be targeted for therapy in colon cancer cells harboring *RAS*-mutant, we evaluated the pharmacological effects of ABT263 and AXIT combination on colon cancer cell lines by measuring the combination indexes (CIs) in the present study. We assayed apoptosis of the cells subject to single or combined treatment of the drugs by flow cytometry analysis of Annexin-V/propidium iodide (PI). We further tested the inhibitory effect on the *in vitro* and *in vivo* growth of the cancer cells. The current data support the pharmacological action mode that the ABT263 and AXIT combination inhibits *RAS*-mutant colon cancer cells by stimulating apoptosis. Our new findings on the pharmacological effects of the ABT263 and AXIT combination on colon cancer may pave a new way to treat this refractory cancer.

## Methods

### Cell culture

Human colon cancer cell lines HCT116, HCT15, and HT29 were purchased from the American Type Culture Collection (Manassas, VA, U.S.A.). The HT29-KRAS cells were generated by introduction of a KRAS (G12D) mutation into HT29 cells through Lentivirus system. In brief, KRAS (G12D) mutation cDNAs were cloned into the pLenti6.2/V5-DEST lentiviral vector (Invitrogen). Lentiviral particles were produced by cotransfection of 293T cells with pLKO.1 constructs and packaging plasmids pMD.G and pCMVR8.91. A Lentivirus was generated; collected and infected cells were selected with 10 μg/ml blasticidin. The positive monoclones were further identified using Sanger sequencing (BioSune, Shanghai, China). HCT15 cells were cultured in RPMI 1640 medium supplemented with 10% fetal bovine serum (HyClone) in the presence of 1% penicillin and streptomycin (HyClone). HCT116 cells were cultured in DMEM with the same supplements. HT29 cells were maintained in McCoy’s 5a medium. The cells were cultured at 37°C under a humidified 95:5 (%; v/v) mixture of air and CO_2_. SW480 cells were grown without CO_2_.

### Drug treatment and cell viability assays

Cells were seeded at a density of 2000–5000 cells/well in 96-well plates one day before the combined drug treatment for 72 h. Cell viability was measured by CellTiter 96 AQueous One Solution from Promega according to the manufacturer’s instructions. Combinations of ABT263 and AXIT were performed as described in the ‘Results’ section, where the CI values were calculated by CalcuSyn software (Version 2; Biosoft). The effect of the combined treatment is dependent on the CI values, where CI < 0.7 is considered synergism; CI = 0.7–0.9, moderate synergism; CI = 0.90–1.10, nearly additive; and CI >1.10, antagonism. ABT263 (Cat. # HY-10087) and axitinib (Cat. # HY-10065) were obtained from Selleck Chemicals.

### Analysis of apoptosis

Cells were seeded in six-well plates and treated with the indicated treatments for 72 h. Cells were harvested and washed by cold PBS, followed by incubation with PI (100 µg/ml) and Annexin V-Alexa Fluor488 conjugate at room temperature for 15 min. Flow cytometry was used to analyze apoptosis with the parameters of 494/518 nm set for Annexin V detection and 535/617 nm for PI.

### Clonogenic assay

HCT116 and HCT15 (1000 cells) were plated in 60-mm dishes and treated with dimethylsulphoxide (control), ABT263 (2 µm), AXIT (1 µm), or a combination of ABT263 and AXIT for 7 days. The cell colonies were stained with crystal violet and counted. The relative number of colonies was calculated by normalization to control as 100%.

### Western blot

For Western blot, proteins were separated in 4–12% SDS-polyacrylamide gels and transferred to polyvinylidene difluoride (PVDF) membranes, followed by blocking in 5% non-fat milk (in Tris-buffered saline plus 0.1% Tween-20). Blots were detected by enhanced chemiluminescence and exposed on X-ray films. The following antibodies from Cell Signaling were used: Cleaved-caspase3, β-actin, Anti-p-Akt (Ser473) (736E11) Rabbit mAb, Anti-p-p44/42 MAPK (Erk1/2) (Thr202/Tyr204) (D13.14.4E) XP® Rabbit mAb, p-β-Catenin (Thr41/Ser45) Rabbit pAb, p-GSK-3β (Ser9) (5B3) Rabbit mAb, AKT, β-catenin, and GAPDH.

### Animal studies

The *in vivo* efficacy of drug treatment was evaluated using C57BL/6 nude mice, which were purchased from National Rodent Laboratory Animal Resources (Shanghai, China). Animals were maintained in pathogen-free conditions, with free access to sterilized food and water. The animal protocol was approved and complied with the guidelines of Institution Animal Care and Use Committee. Cultured HCT116 cells were harvested, suspended in ice-cold PBS, and injected subcutaneously into the flanks. ABT263 was dissolved in saline at a dosage of 20 mg/kg and delivered intravenously twice a week. AXIT was dissolved in sterile water and delivered intravenously at a dosage of 20 mg/kg. Mice were treated with the indicated drugs or vehicles for 5 weeks. Tumor size was measured by calipers every other day and determined by the formula: volume = length × width^2^ × 0.52.

### Statistical analysis

The data are presented as the mean ± SEM. Statistical tests were performed using Microsoft Excel and GraphPad Prism Software version 5.0. Student’s *t*-tests were performed to compare difference of the means between two groups. The statistical significance levels are: * *P* ≤ 0.05, ** *P* ≤ 0.01, *** *P* ≤ 0.001.

## Results

### KRAS-mutant colon cancer cells are selectively sensitive to ABT263 and AXIT combination

**Figure 1:**
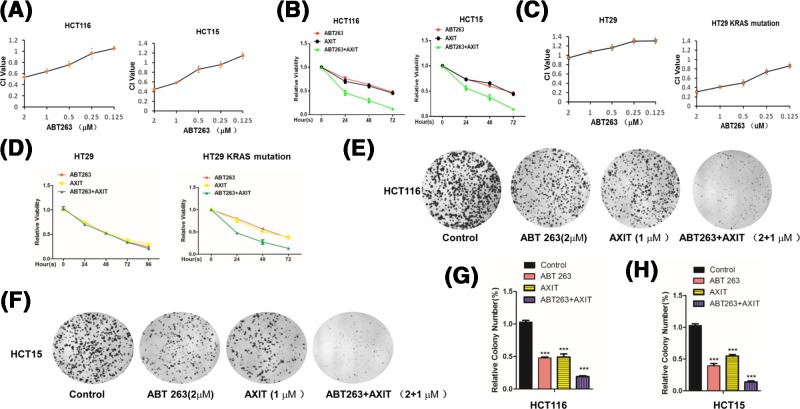
*KRAS*-mutant colon cancer cells are selectively sensitive to ABT263 and AXIT combination treatment (**A**) CIs were calculated for HCT116 and HCT15 cells that had been treated with a combination of AXIT (1 μm) and ABT263 (various concentrations as indicated by the plots). (**B**) Relative viability was measured for HCT116 and HCT15 that were treated with the single drug or combined drugs. (**C**) CIs were calculated for HT29 (left) and HT29-KRAS cells that had been treated with a combination of AXIT (1 μm) and ABT263 (various concentrations as indicated by the plots). (**D**) Relative viability was measured for HT29 (left) and HT29-KRAS (G12D) (right) that were treated with the single drug or combined drugs. (**E**) Clonogenic assay was performed for HCT116 cells that had been treated with single or combination drugs. (**F**) Clonogenic assay was performed for HCT15 cells that had been treated with single or combination drugs. (**G**) Quantification of the clonogenic assay of HCT116 cells shown in (**E**). (**H**) Quantification of the clonogenic assay of HCT15 cells shown in (**F**). (Statistical significance levels are: *** *P* < 0.001).

To evaluate the therapeutic effect of ABT263 + AXIT combination on colon cancer cells, we measured the CIs at the ratio of their IC50’s (Supplementary Figure S1) for various combinations of the two drugs in two colon cancer cell lines, HCT116 and HCT15. We kept a constant concentration (1 μm) of AXIT at its IC50 and variated different concentrations of ABT263 (i.e., 0.125, 0.25, 0.5, 1, and 2 μm), concurrently applying the two drugs to the two colon cancer cell lines. The effect of drug combination is determined by the CI values, with CI < 0.7 being considered synergism; CI = 0.7–0.9, moderate synergism; CI = 0.90–1.10, nearly additive; and CI > 1.10, antagonism. We found that in HCT116 and HCT15 cells, the combination of AXIT (1 μm) + ABT263 (2 μm) showed obvious synergism as CI values were less than 0.7 in both cases (Figure 1A). Since one of the preferable results of drug combination is to achieve synergistic therapeutic effect [16], we decided to use this combination throughout the current study. The cell viability assay showed that the cell growth was inhibited in ABT263 + AXIT combination of these two cells (Figure 1B).

Since both HCT116 and HCT15 carry the G12D *KRAS* mutation [17], we wondered if the observed synergism of the combination was specific to *KRAS*-mutant colon cancer cell lines. We treated HT29, a *KRAS* wild-type cell line, with ABT263 (2 μm) alone, AXIT (1 μm), and ABT263 + AXIT combination, and measured CI values. We found that in HT29 cells, the CI value was almost 1.0, suggesting that the drug effect was nearly additive (Figure 1C). However, the CI value was less than 0.7 in HT29 colon cancer cells exogenously expressing mutant KRAS (Figure 1C). Next, the cell viability was also detected. We observed a much more enhanced killing effect of the ABT263 + AXIT combination compared with the single drug treatment in HT29 *KRAS*-mutant cells (Figure 1D), suggesting that *KRAS*-mutant colon cancer cells may be selectively sensitive to the ABT263 + AXIT combination.

To further compare the cytotoxic effect ABT263 and AXIT, either in combination or alone, on the cancer cells, we performed clonogenic assays for cells that had been treated with ABT263 alone, AXIT alone, or ABT263 + AXIT in combination. Consistently, single drug treatment resulted in fewer formed colonies (Figure 1E,F), and the combination drugs imposed the most severe growth inhibition on the tested cells, suggesting that ABT263 + AXIT in combination may be a potent inhibitor for the growth of *KRAS*-mutant colon cancer cells (*P* < 0.001, Figure 1G,H).

### ABT263 and AXIT combination enhances apoptosis of KRAS-mutant colon cancer cells

**Figure 2:**
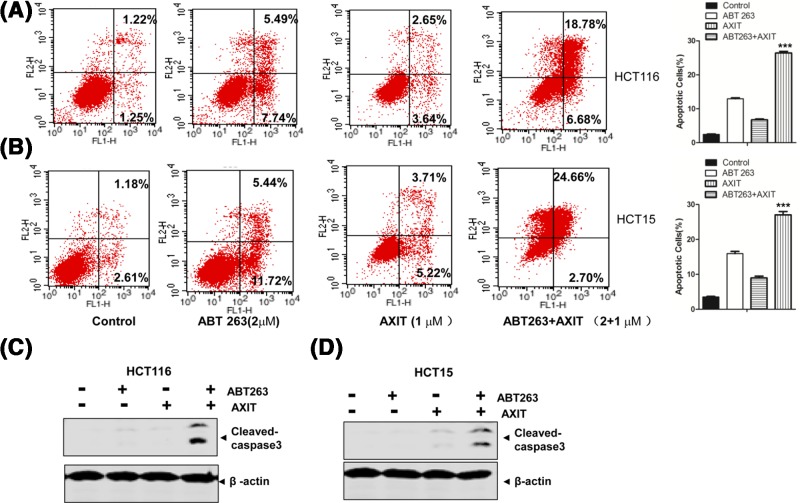
ABT263 and AXIT combination enhances apoptosis of *KRAS*-mutant colon cancer cells (**A**) HCT116 cells treated by single or combination drugs were subject to the Annexin-V/PI apoptosis assay by flow cytometry. The ratios of apoptotic cells to all cells were quantified on the basis of three independent experiments.***P < 0.001. (**B**) HCT15 cells treated by single or combination drugs were subject to the Annexin-V/PI apoptosis assay by flow cytometry. The ratios of apoptotic cells to all cells were quantified on the basis of three independent experiments.***P < 0.001. (**C**) Western blot of Cleaved-caspase3 for HCT116 treated by single or combination drugs. (**D**) Western blot of Cleaved-caspase3 for HCT15 treated by single or combination drugs.

Given the cytotoxic effect of the drugs on the colon cancer cells, we further measured apoptosis of the cells that had been treated with the drugs in combination or each alone. As shown by the Annexin-V/PI apoptosis assay, while ABT263 (2 μm) or AXIT (1 μm) alone could lead to enhanced apoptosis in both HCT116 and HCT15, in the ABT263 + AXIT combination, much higher fractions of the cells underwent apoptosis compared with single drug treatment, suggesting that the combination enhances apoptosis of *KRAS*-mutant colon cancer cells (Figure 2A,B). Consistent with this result, we also observed increased cleaved caspase-3 levels in ABT263 + AXIT combination (Figure 2C,D), suggesting that elevated apoptosis may be responsible for the enhanced cell death observed for ABT263 + AXIT combination in HCT116 and HCT15 cells [18,19].

### AKT and Wnt/β-catenin signaling pathways are down-regulated by ABT263 and AXIT combination in KRAS-mutant colon cancer cells

**Figure 3:**
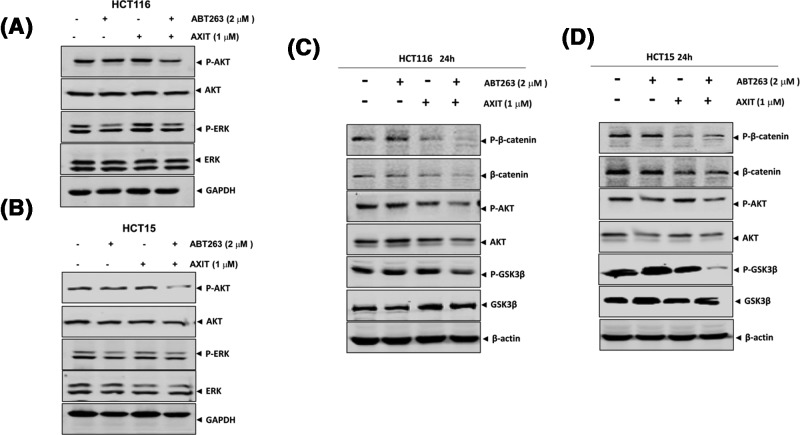
AKT and Wnt/β-catenin signaling pathways are down-regulated by ABT263 and AXIT combination in *KRAS*-mutant colon cancer cells (**A**) Western blots of p-AKT and p-ERK for HCT116 cells treated by single or combination drugs. (**B**) Western blots of p-AKT and p-ERK for HCT15 cells treated by single or combination drugs. (**C**) Western blots of p-β-catenin, p-AKT, and p-GSK3β for HCT116 cells treated by single or combination drugs. (**D**) Western blots of p-β-catenin, p-AKT, and p-GSK3β for HCT15 cells treated by single or combination drugs.

Since ERK is a major downstream effector of RAS signaling, we further tested if the ABT263 + AXIT combination treatment could lead to alteration of ERK. Interestingly, the level of p-ERK was not changed upon the combination treatment in both HCT116 and HCT15 cells (Figure 3A,B). Instead, we found that phosphorylated AKT, β-catenin, and GSK3β levels were slightly down-regulated by the combinatorial drug treatment in both cell lines (Figure 3C,D), suggesting that the PI3K/Akt and Wnt/β-catenin pathways may be involved in targeted oncogene addiction selectively for the *RAS*-mutant colon cancer cells. Taken together, our current data showed that the typical RAS-ERK signaling may be not affected in RAS-mutant colon cancer cells by the combinatorial treatment of ABT263 and AXIT, indicating that this was a MAPK-independent phenotype.

### ABT263 and AXIT combination inhibits *in vivo* growth of KRAS-mutant colon cancer cells

**Figure 4:**
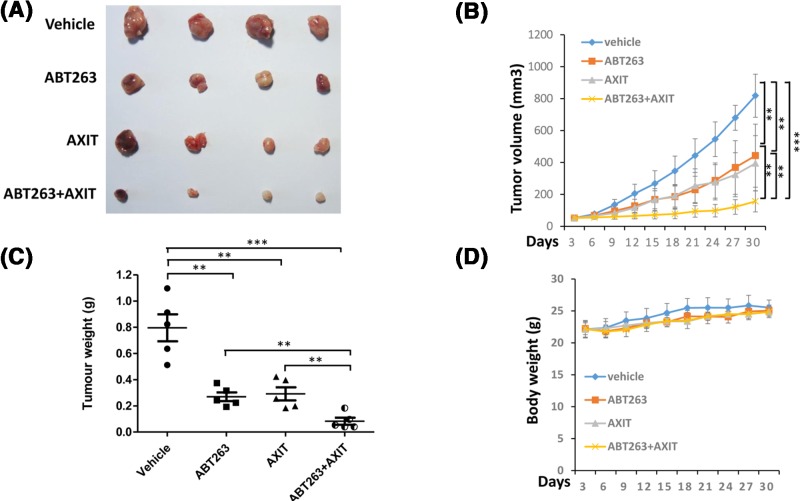
ABT263 and AXIT combination inhibits *in vivo* growth of *KRAS*-mutant colon cancer cells (**A**) Photographs of the xenograft HCT116 tumors that had been treated by single or combination drugs. (**B**) Tumor volume was measured every other day for different drug treatment regiments (i.e., single or combination drugs). Data are presented as mean ± s.d. (**C**) At the end time point, the tumors were harvested and recorded for the weight. Statistical significance levels are: ** *P* < 0.01, *** *P* < 0.001. (**D**) The body weight was detected and recorded.

After we defined the *in vitro* cytotoxic effect of the ABT263 + AXIT combination on colon cancer cells, we further tested its *in vivo* efficacy. We generated xenograft colon tumors in C57BL/6 nude mice and administered ABT263 alone, AXIT alone, or ABT263 + AXIT in combination to the mice every other day. Single drug treatment rendered a significant reduction in tumor size, and the therapeutic effect was the most significant for the combination drugs (*P* < 0.001), demonstrating the potent *in vivo* efficacy of the combination drugs (Figure 4A,B). Measurement of the tumor weight at the end point of the treatment study confirmed the finding as well (*P* < 0.001, Figure 4C). Furthermore, no animals in these studies exhibited significant differences in body weight (Figure 4D), suggesting that all treatments were well tolerated.

## Discussion

In the current study, we address that oncogene addiction can be targeted for therapy in colon cancer cells harboring *RAS*-mutant. We find that the pharmacological effects of ABT263 and AXIT in combination are synergistic on the *RAS*-mutant colon cells. The combination treatment of ABT263 and AXIT can inhibit the *in vitro* and *in vivo* growth of the *RAS*-mutant colon cancer cells. Our discovery provides the important idea of drug combination for treating colon cancer cells.

Synthetic lethality has emerged as a new strategy for treating cancers driven by refractory mutations that are difficult to be targeted, such as *KRAS* mutations. In the therapeutic strategy of targeting oncogene addiction, important nodes in the oncogenic pathways or the ones in cross-talk can be coordinately targeted by existing pharmacological drugs, leading to severe failures in survival and proliferation of the cancer cells. HT29 cells, although KRAS WT, have an activating BRAF V600E mutation, which hyperactivates the MAPK pathway [20]. Our data showed that the proliferation has no big difference in HT29 cells by the combinatorial treatment of ABT263 and AXIT, and the typical RAS-ERK signaling was not affected in RAS-mutant colon cancer cells, indicating that this was a MAPK-independent phenotype. Interestingly, we found that both PI3K/Akt and Wnt/β-catenin pathways were slightly regulated by the combination drugs, hinting for the possibility that the two pathways were important for *RAS*-mutant colon cancer cells. Indeed, Wnt/β-catenin and RAS-ERK pathways can be synergistic and cooperative, whereas both β-catenin and mutant KRAS are stabilized by *APC* loss [1,21–23]. In our case, we found that the level of phosphorylated β-catenin was down-regulated upon treatment of ABT263 + AXIT in combination, suggesting that β-catenin, one factor activated many proliferation genes such as *c-MYC, c-Jun, CCND1, EGFR*, and *CD44* [24,25] could be compromised by the combinatorial drugs. Therefore, we predict that the interaction between the RAS-ERK and Wnt/β-catenin pathways may be accounted for the pharmacological effects of the drugs on the *RAS*-mutant colon cancer cells. To test if this is the case, future work should be dedicated to test (1) if the Wnt level is altered upon treatment, and (2) how the expressions of downstream target genes, such as *c-MYC, c-Jun, CCND1*, and *EGFR*, are changed upon the treatment of combinatorial drugs in both *RAS*-wild-type and -mutant background.

Our study showed that ABT263 and axitininib combination could lead to significant apoptosis. ABT263 is a potent and bioavailable mimetic of BH3 domains that avidly binds Bcl-2, Bcl-xL, and Bcl-W, blocking the interaction of these proteins with pro-apoptotic proteins, leading to apoptosis [26,27]. Furthermore, the combinatorial treatment with axitinib may synergistically enhance the apoptotic process significantly. Axitinib is a tyrosine kinase inhibitor that blocks the activation of VEGFR1, VEGFR2, and VEGFR3 [28]. Given the distinct pharmacological targeting profiles of ABT263 and axitinib, future work should also address a critical question, that is, how to impose the pharmacological effects of these two drugs in *RAS*-mutant cells?

In summary, our current results suggest that oncogene addition targeting may be a feasible way to attack cancers driven by important mutations that are otherwise difficult to be targeted. The distinct features of the known targets of the drug used (i.e., ABT263 and AXIT) and the affected signaling pathways in the RAS-mutant cancer cells add another layer of complexity as far as the detailed mechanism for synthetic lethality in cancer therapy is concerned. Nonetheless, our current results are poised to advance a promising strategy for treating curtain refractory cancers like colon cancer.

## Supporting information

**Figure S1:** The IC50 was detected in HCT116 and HCT15 cells. The concentrations were required to inhibit 50% of cell growth. The results showed that the concentration of ABT-263 and AXIT was almost 2μM and 1μM.

## Abbreviations

CI: combination index
PI: propidium iodide
PVDF: polyvinylidene difluoride
VEGFR: vascular endothelial growth factor receptors
