# Supplementary material for: KRAS-mutant colon cancer cells respond to combined treatment of ABT263 and axitinib
Source: Biosci Rep. 2019 Mar 6;39(3):BSR20181786. doi: 10.1042/BSR20181786 (PMC6400663; doi:10.1042/BSR20181786)
Supplement: Supplementary file 1 [file bsr-39-bsr20181786_Supp1.pdf]

Supporting Figure Legends

Figure S1. The IC50 was detected in HCT116 and HCT15 cells. The concentrations were required to inhibit 50% of cell growth. The results showed that the concentration of ABT-263 and AXIT was almost 2μM and 1μM.

IC50(μM) Drug \ Cell	HCT15	HCT116
ABT263	2.017 ± 0.097	2.024 ±0.328
AXIT	0.876 ± 0.129	1.027 ±0.0.434
